# Commentary: Multiple laser doppler flowmetry probes increase the reproducibility of skin blood flow measurements

**DOI:** 10.3389/fphys.2022.1025905

**Published:** 2022-10-17

**Authors:** Alicia Guigui, Jordan Loader, Alexandre Bellier, Matthieu Roustit

**Affiliations:** ^1^ University Grenoble Alpes, Inserm U1300, Grenoble, France; ^2^ Grenoble University Hospital, Inserm CIC1406, University Grenoble Alpes, Grenoble, France; ^3^ Department of Medical Sciences, Uppsala University, Uppsala, Sweden; ^4^ Computational and Mathematical Biology Team, TIMC-IMAG UMR 5525, CNRS, University Grenoble Alpes, Grenoble, France

**Keywords:** skin blood flux, laser Doppler, reproducibility, repeatability, microcirculation (skin)

Luck et al. (2022) recently reported that the reproducibility of skin blood perfusion measurements with laser Doppler flowmetry (LDF) may be improved by the use of multiple laser Doppler probes at a given region of interest.

Laser Doppler flowmetry is one of several laser-based imaging technologies (e.g., laser Doppler imaging and laser speckle contrast imaging) that can be used to routinely assess skin microcirculation in humans. Additionally, when these laser-based technologies are coupled with tests provocative to the microcirculation (e.g., transdermal iontophoresis of vasoactive substance, local heating or cooling, electrical stimulation *etc.*), microvascular reactivity and/or function can be explored (Cracowski and Roustit, 2020). However, as highlighted by Luck et al*.* (2022), LDF is an older technology using a single-point laser, the reproducibility of which is reduced by the variability in the anatomy of the skin microcirculation; more specifically, the variability in the distribution of capillary loops and arteriovenous anastomoses.

To address the spatial limitation in LDF, Luck et al*.* (2022) used multiple laser Doppler probes concurrently in a single measurement, rather than a single probe as is traditionally implemented. This approach, using multiple, averaged LDF signals *via* so-called “integrative” probes, was demonstrated previously (Tew et al., 2011). Laser Doppler imaging addressed the spatial limitation in LDF by measuring blood flux over a greater area of the skin microcirculation; essentially averaging out the anatomical variations in the microcirculation (Roustit et al., 2010; Puissant et al., 2013). However, laser Doppler imaging is temporally limited. Developed more recently, laser speckle contrast imaging addresses both spatial and temporal limitations of LDF and laser Doppler imaging, respectively, by recording continuous measurements over larger regions of interest. While LDF equipment costs less than laser speckle contrast imaging, this principal advantage is attenuated by the use of multiple laser Doppler probes in a single measurement; raising serious questions about the relevance of the study when considering the use of LDF in practice.

Methodologically, it must be noted that the authors only focused on resting skin blood flux. Overall, baseline flux has little relevance as a biomarker in disease. A striking example of this is in persons with diabetes, where baseline flux may be increased while reactivity/function is usually impaired (Fredriksson et al., 2010). Indeed, the main application of LDF and other laser-based technologies is to assess changes in microvascular reactivity/function throughout the development of disease or in response to an intervention, requiring that they are coupled with a test that challenges (i.e., dilates or constricts) the microvessels in order to provide data of any real value. It is also worth noting that experiments were performed on the non-glabrous skin of the dorsal forearm. Skin microcirculation and, therefore, the reproducibility of LDF measurements is different between the non-glabrous skin assessed by Luck et al*.* (2022) and the glabrous skin evaluated by the majority of previous studies (Cracowski and Roustit, 2020).

Given that all experiments were performed in the same volunteer on the same day, the interday reproducibility of the method used by Luck et al*.* (2022) is unknown. Interday reproduciblity is more relevant than intraday reproducibility when considering patient follow-up or repeated visits in a clinical study. In that context, the necessity to use a semi-permanent marker to ensure replicable placement of the LDF probes does not seem to be suitable for studies requiring follow-up. Additionally, LDF may not be applicable in several conditions, such as surgical interventions, where contact with the wound is generally avoided; further promoting the advantages of non-contact, imaging techniques such as laser speckle contrast imaging.

There were also some errors or inconsistencies that affect the overall quality of the manuscript. While we fully agree that expressing blood flux as cutaneous vascular conductance (CVC) is relevant to account for variations in blood pressure, we question how CVC can be higher than the flux expressed as perfusion units (PU) in Table 3. Indeed, CVC is calculated as the flux (PU) divided by mean arterial pressure (mmHg). Therefore, it is impossible due to the division of a positive number by another positive number ≥1 (CVC = PU/mmHg). In addition, the authors discuss the “mean statistical power” for the intraclass correlation coefficient. The statistical power is the probability that a test correctly rejects the null hypothesis when the alternative hypothesis is true. It is useful to calculate sample size before the study begins, based on one hypothesis. Whether “mean power” is useful is not clear. However, it would be useful to have an indication of the precision of the estimates from this sample size by providing 95% confidence intervals. Although the authors stated that these were calculated, we were unable to find them.

There is also confusion within the manuscript regarding imaging techniques, “laser-Doppler speckle contrast imaging” does not exist. Laser speckle contrast imaging is not based on the Doppler effect, these are two distinct techniques. There is also some inconsistencies in the terminology (flow versus flux). Indeed, these laser-based techniques do not provide a measure of *flow* (i.e., volume of fluid per unit time), but arbitrary PU, often referred to as *flux*, which does not permit direct comparisons between technologies.

Overall, Luck et al. (2022) is one of many studies that has introduced a questionable, additional technique using an inferior technology into a field that’s already over-saturated with unstandardized methodologies. Research resources would be better directed to refining techniques that are already known to be superior.
